# Addressing Inequalities Toward Inclusive Governance for Achieving One Health: A Rapid Review

**DOI:** 10.3389/fpubh.2021.755285

**Published:** 2022-01-20

**Authors:** Eliudi S. Eliakimu, Linda Mans

**Affiliations:** ^1^Health Quality Assurance Unit, Ministry of Health, Community Development, Gender, Elderly and Children, Dodoma, Tanzania; ^2^An Independent Consultant and Researcher in Support of Healthy People and a Healthy Planet, Manskracht, Nijmegen, Netherlands

**Keywords:** inequality, One Health (OH)-approach, Sustainable Development Goals, COVID-19, climate change, inclusive governance, inclusive development, zoonotic diseases

## Abstract

Sustainable development goals (SDGs) adopted in 2015 are geared toward sustainable development through various pathways, one being reducing inequality as covered in SDG 10. Inequalities are a threat to health and wellbeing of populations and a planet Earth in which we live. This rapid review aims to identify key issues that are likely to exacerbate inequalities around the six SDGs directly related to One Health, which are SDG 3, 6, 11, 13, 14 and 15, and suggest some actions that may help to address them using inclusive governance taking into account the coronavirus disease of 2019 (COVID-19) pandemic. Informed by the literature on SDGs and using the “*inclusive development concept*” by Gupta and Vegelin, literature search was done in Google Scholar, PubMed Central, as well as, searching of references in the relevant articles identified using search terms from the six SDGs that are directly related to One Health. In the context of the SDGs, in order to achieve One Health through inclusive governance, and tackle inequalities, the following needs to be considered and addressed: increasing number of armed conflicts; ongoing COVID-19 pandemic; ensuring availability of water and sanitation facilities; improving city and urban areas planning to cope with climate change; improving governance arrangements for addressing climate change factoring gender and human rights; multisectoral planning for conservation of oceans, seas, and marine resources; balancing trade regulation of wildlife trade with conservation efforts; need for a research collaborative involving experts from environmental sciences, wildlife, agriculture and human health to study and develop scientific evidence on contribution of changes in land use practices to occurrence of zoonotic diseases; and need of a legislation for promoting animal welfare to protect public health. Also, inclusion of people with disabilities in the use of digital technologies is critical.

## Introduction

The United Nations agenda for sustainable development to transform our world by 2030, articulated the 17 Sustainable Development Goals (SDGs). Targets for the SDG 10 which aims to “*reduce inequality within and among countries*” focus on five key actions areas as follows: income inequality and growth; financial and trade issues; migration; social, economic and political inclusion of all; and representation and voice of developing countries in global international economic and financial institutions (1). Six of the 17 SDGs are related to One Health directly, which are SDG3-*ensure healthy lives and promote well-being for all at all ages*; SDG 6-*ensure availability and sustainable management of water and sanitation for all*; SDG11- *make cities and human settlements inclusive, safe, resilient and sustainable*; SDG13-*take urgent action to combat climate change and its impacts*; SDG14-*conserve and sustainably use the oceans, seas and marine resources for sustainable development*; and SDG15-*protect, restore and promote sustainable use of terrestrial ecosystems, sustainably manage forests, combat desertification, and halt and reverse land degradation and halt biodiversity loss* (1, 2).

The progress made in the implementation of the SDGs since their adoption in 2015 has decreased in 2020 due to “*a decline driven to a large extent by increased poverty rates and unemployment following the outbreak of the COVID-19 pandemic; which has impacted all three dimensions of sustainable development: economic, social, and environmental*” (3). One Health approach defined as **“***a collaborative, multisectoral, and transdisciplinary approach—working at the local, regional, national, and global levels—with the goal of achieving optimal health outcomes recognizing the interconnection between people, animals, plants, and their shared environment;*”(4) is an important avenue for ensuring that the world we leave is safe and healthy. To achieve this aim requires taking a governance approach that addresses the needs of and takes everyone on board (i.e., inclusive governance).

The “*inclusive development concept*” by Gupta and Vegelin (5) connected with the work by Veronica Ormea (2) provides a useful framework for analyzing drivers of inequalities that can be addressed within the lens of inclusive governance toward One Health withing the SDGs era and beyond. Therefore, this rapid review aims to analyze key drivers of inequalities around the six SDGs directly related to One Health and suggest some actions that may help to address it using inclusive governance taking into account the coronavirus disease of 2019 (COVID-19) pandemic.

## Methods

Informed by the literature on SDGs and using the “*inclusive development concept*” by Gupta and Vegelin (5), literature search for this rapid review was done using the search terms from the six SDGs that are directly related to One Health. Searches were done in Google Scholar, PubMed Central (from 07^th^ to 13^th^ July 2021), as well as, searching of references in the relevant articles published in the last five years (2017–2021) and identified using the following search terms: climate change; inequality and climate change; sustainable cities; inequality in availability of water and sanitation facilities; sustainable use of oceans, seas and marine resources; land degradation and biodiversity loss; sustainable use of terrestrial ecosystems; inequality in health and well-being. One of the authors (ESE) did the first round of literature search, qualitative data extraction, and drafted the results. Then, shared with the co-author (LM) who independently read the paper and looked at the data extraction for relevance and improvements/modifications. Where there were differences the two authors discussed and reached consensus on the best result.

## Results

The initial literature search obtained 35 relevant papers to the study aim; and in subsequent revision of the manuscript, three references were added making a total of 38 papers. Table 1 shows the details of the papers and the SDGs that are addressed by the papers.

**Table 1:** Retrieved literature.

**References**	**Study Title**	**SDGs addressed**
Strand and Hegre (6)	Trends in Armed Conflict, 1946–2020.	Ensure healthy lives and promote well-being for all at all ages
Akseer et al. (7)	Women, children and adolescents in conflict countries: an assessment of inequalities in intervention coverage and survival.	
Dickinson et al. (8)	Structural Racism and the COVID-19 Experience in the United States.	
Cuevas et al. (9)	Discrimination and systemic inflammation: A critical review and synthesis.	
Shiau et al. (10)	The Burden of COVID-19 in People Living with HIV: A Syndemic Perspective.	
Singer et al. (11)	Syndemics and the biosocial conception of health.	
Amimo et al. (12)	A review of prospective pathways and impacts of COVID-19 on the accessibility, safety, quality, and affordability of essential medicines and vaccines for universal health coverage in Africa.	
Shankar-Hari et al. (13)	Association Between Administration of IL-6 Antagonists and Mortality Among Patients Hospitalized for COVID-19: A Meta-analysis.	
Cullinan (14)	Finally, Therapeutics for Severe COVID-19 – But They Come With Hefty Price Tags.	
Cullinan (15)	Roche Suspends Patents on Tocilizumab in LMICs After WHO Recommends it as Treatment for Severe COVID-19.	
Local Burden of Disease WaSH Collaborators (16)	Mapping geographical inequalities in access to drinking water and sanitation facilities in low-income and middle-income countries, 2000-17.	Ensure availability and sustainable management of water and sanitation for all
Gwenzi (17)	Leaving no stone unturned in light of the COVID-19 fecal-oral hypothesis? A water, sanitation and hygiene (WASH) perspective targeting low-income countries.	
Hobbie and Grimm (18)	Nature-based approaches to managing climate change impacts in cities.	Make cities and human settlements inclusive, safe, resilient and sustainable
Lin et al. (19)	Integrating solutions to adapt cities for climate change.	
Yahia et al. (20)	Effect of urban design on microclimate and thermal comfort outdoors in warm-humid Dar es Salaam, Tanzania.	
Seddon et al. (21)	Understanding the value and limits of nature-based solutions to climate change and other global challenges.	
Patel (22)	Preventing COVID-19 Amid Public Health and Urban Planning Failures in Slums of Indian Cities.	
Lloyd et al. (23)	A Global-Level Model of the Potential Impacts of Climate Change on Child Stunting via Income and Food Price in 2030.	Take urgent action to combat climate change and its impacts
Janssens et al. (24)	Global hunger and climate change adaptation through international trade.	
Andrijevic et al. (25)	Overcoming gender inequality for climate resilient development.	
Aryal et al. (26)	Climate risks and adaptation strategies of farmers in East Africa and South Asia.	
Talukder et al. (27)	Health impacts of climate change on smallholder farmers.	
Bezgrebelna et al. (28)	Climate Change, Weather, Housing Precarity, and Homelessness: A Systematic Review of Reviews.	
Baker et al. (29)	Infectious disease in an era of global change.	
Beyer et al. (30)	Shifts in global bat diversity suggest a possible role of climate change in the emergence of SARS-CoV-1 and SARS-CoV-2.	
Rocque et al. (31)	Health effects of climate change: an overview of systematic reviews.	
Bruno et al. (32)	Climate change threatens the world's marine protected areas.	Conserve and sustainably use the oceans, seas and marine resources for sustainable development
Carr et al. (33)	The Aichi Biodiversity Targets: achievements for marine conservation and priorities beyond 2020.	
Grip and Blomqvist (34)	Marine spatial planning: Coordinating divergent marine interests.	
Aguirre et al. (35)	Illicit Wildlife Trade, Wet Markets, and COVID-19: Preventing Future Pandemics.	Protect, restore and promote sustainable use of terrestrial ecosystems, sustainably manage forests, combat desertification, and halt and reverse land degradation and halt biodiversity loss
Morcatty et al. (36)	Online trade in wildlife and the lack of response to COVID-19.	
Shivaprakash et al. (37)	Mammals, wildlife trade, and the next global pandemic.	
Roe et al. (38)	Beyond banning wildlife trade: COVID-19, conservation and development.	
McNamara et al. (39)	COVID-19, Systemic Crisis, and Possible Implications for the Wild Meat Trade in Sub-Saharan Africa.	
Wikramanayake et al. (40)	Evaluating wildlife markets for pandemic disease risk.	
Wikramanayake et al. (41)	A tool for rapid assessment of wildlife markets in the Asia-Pacific Region for risk of future zoonotic disease outbreaks.	
Plowright et al. (42)	Land use-induced spillover: a call to action to safeguard environmental, animal, and human health.	
Whitfort (43)	COVID-19 and Wildlife Farming in China: Legislating to Protect Wild Animal Health and Welfare in the Wake of a Global Pandemic.	

Key drivers of inequality are presented as per the six SDGs that are directly related to One Health.

### Ensure Healthy Lives and Promote Well-Being for All at All Ages

As we move forward with the SDGs era, armed conflicts continue to affect various countries in various regions globally affecting health and well-being of populations involved. For instance, in 2020 there were 56 active conflicts of which eight were wars, an increase from seven wars in 2019 and six wars in 2018 (6). Conflicts also affect coverage of health interventions to vulnerable populations especially women, children and adolescents leading to health inequalities in conflict affected countries compared to countries without conflicts (7).

The current COVID-19 pandemic has worsened the health and well-being of global populations in many ways including a high mortality, in which as per the World Health Organization (WHO) COVID-19 dashboard “*as of 2:24pm CEST, 13 July 2021: there have been 186,821,815 confirmed cases of COVID-19, including 4,038,342 deaths, reported to WHO*” (44). The COVID-19 has been shown to exacerbate inequalities even in high income countries with racial disparities in outcomes, which calls for a more research to gain an insight on what makes such inequalities to continue so that the world can better prepare for future pandemics as well as addressing the gaps in the ongoing pandemic (8). The epidemiological role of discrimination is found to be important. Cuevas et al. show that experiences of discrimination, both acute and chronic, can dysregulate immune function, characterized by elevated levels of inflammation, i.e., worsening people's health and well-being (9). Also, the COVID-19 pandemic is likely to add more burden to countries with high burden of Human Immunodeficiency Virus (HIV) infections in terms of social and psychological factors. In this case it requires more attention employing the “*syndemic approach*” in order to uncover vulnerabilities and design relevant strategies to protect the affected populations (10, 11). The pandemic has also impacted on the global supply chain for health commodities in an unprecedented way. In order to support low-and middle- income countries (LMICs) especially those in the African continent in a way that will ensure availability of essential health commodities that will ensure continuity of essential services and address the needs for care of COVID-19 patients, it has been proposed that support need to be informed by data on “*comparative risk assessment*” (12).

Apart from great efforts in the area of research and innovation to combat the COVID-19 pandemic, global, regional and country cooperation and solidarity efforts are needed to enable deployment of discoveries to save lives of people who need them. For instance, in a recent meta-analysis of 27 trials which “assessed the efficacy of Iterleukin-6 (IL-6) antagonists in patients hospitalized for COVID-19,” it was found that “administration of IL-6 antagonists (Tocilizumab and Sarilumab), compared with usual care or placebo, was associated with lower 28-day all-cause mortality” (13). The World Health Organization (WHO) immediately (on 06^th^ July 2021) went on to recommend the use of the medicines for COVID-19 patients; however, the biggest challenge to health systems in low-and middle- income countries (LMICs) is a high price of the medicines (14). This means that high income countries and international organizations must find ways that will ensure the medicines are accessible to poor countries in which the number of COVID-19 cases and deaths is increasing and also, they lack access to COVID-19 vaccines. In signs of enhanced commitment to save human life, the Swiss pharmaceutical company Roche (a day after WHO recommended the use of the medicines) announced that it has suspended its patent rights on the medication tocilizumab in LMICs for the duration of the pandemic, an action that will contribute to its accessibility to people in need in LMICs (15). Therefore, other manufacturing companies and high-income countries should also do the same to support LMICs to access the live saving health commodities including the COVID-19 vaccines.

### Ensure Availability and Sustainable Management of Water and Sanitation for All

Availability of water and sanitation facilities in LMICs has increased in the past two decades, however inequalities in access still persist at subnational levels with more access in urban areas (16). Concerted efforts to address this inequality in access to water and sanitation facilities are needed given the ongoing COVID-19 pandemic (17).

### Make Cities and Human Settlements Inclusive, Safe, Resilient and Sustainable

Cities in LMICs are growing rapidly and due to inadequate resources, they are inadequately prepared to handle effects of climate change. Implementation of nature-based solutions have been shown to have potential for improving living conditions in cities (18); and in particular due to the fact that they are low cost and widely accessible (19). A study that assessed the effect of urban design in relation to the nature-based solutions in Dar es Salaam, Tanzania, provided some insights that can be used to improve living conditions and address effects of climate change which require city planners and architects to take them on board; an indication of how inclusive we should be in terms of expertise involvement if we are to achieve One Health and be able to tackle such global health challenges as climate change (20). In order to realize the full potential of nature-based solutions, there is a need for further research and to ensure that its implementation take into account a systems-thinking framework (21). Also, inadequate planning of cities in LMICs as manifested by people living in slum areas affects implementation of interventions to improve well-being of the people. The challenges observed in addressing the ongoing COVID-19 pandemic are further enlightening us on the need for improving planning of cities and other urban areas in order to allow implementation of public health measures necessary for combating the current and future epidemics (22).

### Take Urgent Action to Combat Climate Change and Its Impacts

The threat of climate change to the world we live are many, hence, threatening our health and well-being. Effects of climate change to the nutrition of children under 5 years are likely to be greater in rural areas in which income of the people and prices of food are likely to affect availability of food to children (23). Also, climate change affects food accessibility to populations and exacerbates poverty and inequality. In addressing the challenge of famine, adaptation using adjustments in international trade such as reducing tariffs and barriers at organizational level as well as infrastructural barriers have been shown to have potential for addressing food shortages (24). Along these efforts, in order to address inequalities and ensure inclusiveness, there is a need for ensuring that gender equality is part and parcel of the interventions to address effects of climate change (25). Also, small-scale farmers are affected by climate change. For example, a study by Aryal and colleagues, has reported effects of climate risks to farmers in East Africa and South Asia in which they found an inadequate capacity to deal with effects of climate change which was further compromised with inadequate governance (26). Health effects of climate change to small-scale farmers have been reported to range from “*communicable diseases; non-communicable diseases; mental health; and occupational health, safety and other health issues*” (27). These effects require actions to be taken at country level and global level (27). Taking into account that climate change also affects disproportionately people without housing, Bezgrebelna and colleagues have suggested for application of a “*Human Rights-Based Approach*” in designing of interventions to address this situation (28). We note that health impacts of climate change are enormous and may increase as global warming continues. According to Baker et al. climate change, rapid urbanization and changing land-use patterns will increase the risk of disease emergence in the coming decades. Climate change, in particular, may alter the range of global pathogens, allowing infections, particularly vector-borne infections, to expand into new locations (29). Climate change may have played a key role in the evolution or transmission of SARS-CoV-1 and SARS-CoV-2 (30). Therefore, it is important to continue researching on its dynamics so that we can understand vulnerabilities and hence address the inequalities resulting from its effects (31).

### Conserve and Sustainably Use the Oceans, Seas and Marine Resources for Sustainable Development

Sustainability of oceans, seas and marine resources continues to be affected by climate change, and if the global efforts to address climate change are not effectively harnessed, effects of climate change will be huge by 2050 (32). With the economic role attached to these resources by countries and populations, it is extremely important now to take collective actions that will improve conservation interventions for our current and future well-being (33). In planning process for addressing these challenges, the “*Marine spatial planning*” as described by Grip and Blomqvist, offers a multisectoral planning process “*for coordinating different marine interests and balancing the use, protection and conservation of marine areas and space with its resources, biodiversity and ecosystem services*” (34). By multisectoral planning, it will ensure that the needs of those who depend on marine resources for their livelihood are taken into account.

### Protect, Restore and Promote Sustainable Use of Terrestrial Ecosystems, Sustainably Manage Forests, Combat Desertification, and Halt and Reverse Land Degradation and Halt Biodiversity Loss

As part of addressing the COVID-19 pandemic, need for considering control of business of live wild animals and also eating practices in terms of regulating wildlife markets was noted (35, 36). However, researchers have suggested that wildlife trade is a source of income for the poor people therefore indiscriminate banning may exacerbate poverty and may also not be sustainable in the longer term in addressing risk of zoonotic diseases to humans. Therefore, balancing trade regulation with conservation efforts will be a better approach to allow those who depend on it to earn their living and to contribute to their economic development (37–39). In order to better address this, it has been suggested to find ways of assessing the markets for risk of transmission (37, 40). Wikramanayake and colleagues have developed a tool based on the situation in the South East Asia which can be used for this purpose and probably applicable to other global regions as well (40, 41). The need to complement such regulatory efforts with strategies to improve use of land in order to minimize spread of zoonotic diseases from animals to humans has been emphasized (42). Plowright and colleagues have called for a collaborative effort involving experts from environmental sciences, wildlife, agriculture and human health to study and develop scientific evidence on contribution of changes in land use practices to occurrence of zoonotic diseases (42). Also, the authors have pointed out that findings from such collaborative research on land use will “*help to understand and show how investments in landscape conservation provide returns for human health, climate change, international trade, sustainable development, environmental justice, and other policy issues associated with human well-being*” (42). The debate about the origin of the severe acute respiratory syndrome coronavirus 2 (SARS-CoV-2), the virus that causes COVID-19, has reminded us about the health and welfare of animals. A paper by Whitfort has described the gaps in China in terms of the lack of a legislation for promoting animal welfare, which is key in protecting public health, and that moving forward “*decisions about animal welfare law and policy require a global vision*” (43).

## Discussion

In the context of the SDGs, in order to achieve One Health through inclusive governance, and tackle inequalities, the following need to be addressed: armed conflicts; ongoing COVID-19 pandemic; availability of water and sanitation facilities; improving city and urban areas planning; improving governance arrangements for addressing effects of climate change factoring gender and human rights; multisectoral planning for conservation of marine resources; balancing trade regulation wildlife trade with conservation efforts; research collaborative involving experts from environmental sciences, wildlife, agriculture and human health to study and develop scientific evidence on contribution of changes in land use practices to occurrence of zoonotic diseases; and need for a legislation for promoting animal welfare to protect public health.

As Sachs and colleagues have put it clearly in the SDGs report of 2021, “*digital technologies have played a critical role in sustaining social services, payments, schooling, and health care during the lockdowns, and in enabling working from home to be effective for many occupations. The importance of digital applications underscores the vital importance of universal access to broadband services as key to social inclusion, economic opportunity, and public health*”(3). However, currently there is no adequate research evidence about digital technologies and disability that will allow inclusion and participation of people with disabilities in the digital era (45). Strengthening research in this area is of paramount importance in order to ensure that people with disabilities are not sidelined in the digital revolution. Having research evidence that is contextualized to country context is critical, taking example of the study by Lin and colleagues in China (46). Also, there is a need to delve further into the interplay between power and inequalities taking into account sources, and forms of power in people's everyday life (47).

In order to achieve health and well-being in the context of SDGs improving accountability and human rights is imperative (48). The efforts to protect the nature from human destruction requires governments to: ensure financial resources reach the local people; take into account human rights issues; and involve local people in conservation efforts and fostering partnerships with stakeholders (38). Moving forward, there is also a need to strengthen country capacities (with global coordination) for conducting research on health inequalities (49).

In terms of addressing risk of zoonotic diseases epidemics and pandemics, multisectoral collaboration is essential. As also stated in the “tripartite zoonoses guide” (50), that countries need to strengthen “*multisectoral communication, coordination, and collaboration*” in order to better address current and future pandemics from zoonotic pathogens. A proposal by Frieden and colleagues on a measure aiming at instituting accountability in handling outbreaks offers an avenue of accountability at country level and global level to avoid future effects we are witnessing currently with the COVID-19 pandemic. The proposed measure has a target of “detection within 7 days; notification, investigation, and initiation of response within 1 day; and establishing effective control measures within 7 days” (51). As, put forward by Weible and colleagues, such issues that are at a level of a global policy challenge such as COVID-19 deserves to be addressed through “*transnational administration”* (52).

## Conclusion

Inequalities affect health and well-being of populations globally, regionally and at country level. Inclusive approach to governance is a potential way to take onboard everyone and hence reduce inequalities. The ongoing COVID-19 pandemic has exacerbated inequality and its drivers, hence, threatening to strain our efforts to address inequalities through One Health approach. While, we have identified some key strategies to address inequalities in relation to the six SDGs directly related to One Health, they will need to be supported by strong policies at global, regional and country level learning from policy sciences. It also requires strengthened country capacities for conducting research on health inequalities. We therefore, call for inclusive governance in addressing inequalities to achieve One Health.

## Author Contributions

EE conceptualized the manuscript, wrote the first draft of the manuscript, and contributed to review of the subsequent versions of the manuscript. LM contributed to the writing of the manuscript and review of the manuscript. Both authors have read and approved the final manuscript.

## Conflict of Interest

The authors declare that the research was conducted in the absence of any commercial or financial relationships that could be construed as a potential conflict of interest.

## Publisher's Note

All claims expressed in this article are solely those of the authors and do not necessarily represent those of their affiliated organizations, or those of the publisher, the editors and the reviewers. Any product that may be evaluated in this article, or claim that may be made by its manufacturer, is not guaranteed or endorsed by the publisher.
